# Assessing the Impact of a Longitudinal Student-Run Medical Spanish Course in a California Doctor of Medicine (MD) Program

**DOI:** 10.7759/cureus.105015

**Published:** 2026-03-10

**Authors:** Layla Ali, Niloufar S. Tehrani, Lucas Owens, Shaumik Patil, Jon-Richard Sandell-Zanipatin, Samuel Salib, Adam Ali, Rebecca Phelan, Carol L Conrad-Forrest

**Affiliations:** 1 Medicine, California Northstate University College of Medicine, Elk Grove, USA; 2 Otolaryngology - Head and Neck Surgery, California Northstate University College of Medicine, Elk Grove, USA; 3 Obstetrics and Gynecology, California Northstate University College of Medicine, Elk Grove, USA

**Keywords:** medical education, spanish, spanish course, spanish health education, spanish-speaking patients

## Abstract

Introduction

Spanish is the most commonly spoken non-English language in the United States (US), and language discordance remains a major barrier to patient-physician communication and health equity. While many US medical schools now offer Medical Spanish curricula, limited evidence exists evaluating whether greater participation translates into improved perceived communication competency. This pilot study examined whether attendance in a student-run medical Spanish course was associated with higher overall Spanish comfort and clinical communication confidence.

Methods

Medical students at a California doctor of medicine (MD) program enrolled in a 10-session, in-person, systems-based medical Spanish course combining vocabulary instruction with clinical role-play. Participants completed pre- and post-course surveys assessing overall Spanish comfort and comfort interacting with Spanish-speaking patients using a five-point Likert scale. Linear regression models evaluated associations between session attendance and comfort outcomes, with secondary adjustment for preferred learning modality (listening, speaking, or writing).

Results

Fourteen students completed the surveys. Increased session attendance was significantly associated with higher overall Spanish comfort (β = 0.16 points per session, p = 0.026; R² = 0.37), demonstrating a dose-response relationship. A positive trend was observed for clinical-interaction comfort (β = 0.13), though this did not reach statistical significance (p = 0.16).

Discussion

Participation in a student-run medical Spanish curriculum was associated with significantly higher perceived overall Spanish competency and positive trends toward improved clinical communication confidence. These findings support the feasibility and health-equity value of structured language training initiatives and highlight the role of student-led programs in improving culturally responsive care for Spanish-speaking populations. Larger studies incorporating objective proficiency assessments are warranted.

## Introduction

The patient-physician relationship is arguably one of the most important aspects in the effective practice of medicine, and essential to that relationship is clear communication. Additionally, while interpreters can bridge language gaps, patients report more satisfaction and even confidence when physicians have the ability to speak in both English and Spanish [1]. According to the 2019 Census, Spanish is the second most popular spoken language, following English in the United States (US). Consequently, medical Spanish is becoming an increasingly popular curriculum to implement in medical schools across the US. In a national survey of US medical schools, an estimated 66% of medical schools surveyed reported offering a medical Spanish curriculum to their students. Additionally, of those who reported having no curriculum, 32% reported a plan to incorporate this into the curriculum in the next two years [2].

Not only is this curriculum more popular, but it is also showing increased necessity. In studies of children with medical complexity who have a Spanish-speaking caregiver, communication challenges were described as having detrimental consequences for their long-term health outcomes and recovery. Bilingual coordinators helped lessen these consequences [3], demonstrating the necessity of bilingual individuals in healthcare.

Unfortunately, there is a lack of bilingual medical interpreters who can fulfill this need [4]. According to the experiences of Spanish-speaking patients at an academic medical center, language barriers and social determinants of health frequently compounded one another, creating additional obstacles to accessing care [4]. Spanish-speaking Latino participants in the study were more likely to have lower income, lower levels of formal education, reduced employment, and less access to private insurance compared with English-speaking respondents [4]. These structural and linguistic challenges can interact to limit patients' ability to navigate the healthcare system, communicate effectively with providers, and obtain appropriate services, thereby contributing to disparities in healthcare access and utilization [4]. Therefore, the paper suggested that academic medical centers may need multifaceted interventions that improve the availability of bilingual staff and interpreters and also address caregivers' social and informational needs [4].

Language barriers reflect a general pattern among the Latino community, where there is a high prevalence of medical mistrust among both English and Spanish-speaking Latino parents of adolescents. The shared mistrust was of how healthcare systems view and treat people of their racial/ethnic background [5]. Due to the intertwined nature of culture and language, an increase in bilingual healthcare providers who demonstrate cultural competency could then mitigate this widespread mistrust.

In implementing a medical Spanish curriculum, it is important to properly assess the Spanish-speaking ability of its students. There is considerable variation in perceived Spanish proficiency versus standardized patient (SP) and faculty-rated fluency in objective structured clinical examination (OSCE) scenarios; SP and faculty ratings were each higher than the student self-rated pre-course fluency (p < 0.001) [6]. Loyola University adopted the Spanish Bilingual Medical Student Certification, which utilized online assessments such as the Cultural and Linguistic Assessment (CCLA), Qualified Bilingual Staff Assessment (QBSA), and Spanish Objective Structured Clinical Exam (SOSCE) to test their student's proficiency in medical Spanish [7].

Additionally, creating a student-run curriculum may prove the advantage of higher attendance, and more comfort with instructors, and has been proven feasible before. On the other side, this method may lack professional oversight and have no certifications [8].

Finally, second language acquisition ability proves to have a steady decline following around 18 years of age [9]. Therefore, the earlier that the Spanish curriculum is integrated into medical education, the more robust the effect will be. This highlights the importance of offering a medical Spanish curriculum in more US medical schools. We examine the feasibility of the implementation of an in-person curriculum in one of the areas that need bilingual physicians the most, California. There are certain advantages and disadvantages to how this curriculum will be presented. The virtual curriculum allows for self-pacing, ease of access, and lower cost. However, an in-person curriculum allows for more structure, immediate feedback, and potential improvement in accent and dialect. We chose to implement an in-person curriculum due to students already being present most days and a perceived higher level of commitment to the course versus signing up for a virtual curriculum.

The purpose of this study is to evaluate the subjective competency between medical Spanish session attendance and composite comfort, with secondary outcomes evaluating pre- and post-course objective medical Spanish knowledge. The main hypothesis of this study is that greater attendance and course completion would be associated with higher self-rated comfort and improved exam scores.

## Materials and methods

Study population

All first- or second-year medical students at California Northstate University College of Medicine, Elk Grove, USA, between the ages of 18 and 35 who expressed interest in learning medical Spanish were eligible to participate in the study. Sixty students filled out the pre-course assessment to participate in the course. Exclusion criteria include participants who self-reported that they learned Spanish as a first language. No students met the exclusion criteria. Inclusion criteria include all participants who enroll in the advanced medical Spanish course.

This project was designed as a pilot educational evaluation to estimate the direction of change in comfort and knowledge of Spanish and to assess feasibility (i.e., participation), rather than to provide a definitive, fully powered efficacy estimate. Accordingly, we used a convenience sample consisting of all learners who participated during the study period and completed the surveys (n = 14).

All first- and second-year medical students at California Northstate University College of Medicine were informed of the course through institutional announcements and invited to participate. Fourteen students enrolled in the course and completed the post-course comfort survey (n = 14). Of these, four students completed both the pre- and post-course objective knowledge assessments and were included in the paired analysis of objective knowledge gains. Attendance data were recorded for all participants and used in regression analyses examining associations between the number of sessions attended and composite comfort scores.

Course design

Curricular Design and Structure

We developed a longitudinal, student-run medical Spanish course for pre-clinical medical students organized by body system. The curriculum consisted of an introductory session on basic clinical encounter structure in Spanish (“anatomy of a visit”), followed by organ-system-based sessions (e.g., constitutional and intake, hematology, musculoskeletal/integumentary, HEENT (head, eyes, ears, nose, throat), metabolic/endocrine, gastrointestinal, neurologic/psychiatric, cardiopulmonary, and genitourinary/reproductive). This schedule can be found in Appendix A. Each session included pre-class preparation (e.g., vocabulary sheets, brief readings, or Anki decks), a large-group didactic component, and small-group practice activities aligned with that week's system. In total, the course was meant to include 30 minutes of preparation time to complete pre-course materials, along with a 1.5-hour session, for a total of 10 two-hour sessions over the course of the school year.

Learner Grouping by Proficiency

After the initial large-group lecture, learners would sort into their tested proficiency levels: beginner, intermediate, and advanced. Beginner groups focused on foundational grammar (e.g., “to be” in a temporary vs. permanent state (ser vs estar), regular verb conjugations) and high-frequency phrases needed for basic introductions and histories, using structured practice with a core textbook and worksheet-based exercises. Intermediate and advanced groups applied system-specific vocabulary to simulated clinical scenarios, translation tasks, and brief written or oral responses (e.g., conducting a focused review of systems).

Instructional Team and Use of Translators

Sessions were co-led by medical student instructors with prior Spanish training and volunteer professional or community translators. Translators attended class sessions to model culturally and linguistically appropriate phrasing, answer real-time language questions from learners, and reinforce correct usage during role-plays. Instructors used a standardized planning checklist to ensure each session included (1) a brief background mini-lecture, (2) vocabulary introduction (Appendix B), (3) interactive small-group practice (e.g., paired interviews, OSCE-style prompts) (Appendix C), (4) optional homework or Anki review, and (5) a brief quiz or formative assessment when applicable (Appendix D).

Session Flow

During the large-group portion, instructors introduced key phrases and modeled a Spanish-language clinical encounter (e.g., greeting, confirming patient identity, clarifying language preference, eliciting the chief concern, and closing the visit). Learners then transitioned to proficiency-based groups for targeted practice. In small groups, students alternated roles (physician, patient, observer) and used printed worksheets and vocabulary lists to complete guided dialogue, symptom-focused questioning, and brief written translations.

Study design

This study was approved according to IRB protocol 2407-02-159. First, all students who are interested in learning medical Spanish were given a 15-question assessment to assess their Spanish-language knowledge and determine their course level (Appendix E). This assessment was generated by the directors of the medical Spanish course and was based on a similar assessment that the formal medical interpreters were given in their own training.

The objective knowledge assessment consisted of a 15-item multiple-choice and short-response examination developed by course directors based on high-frequency clinical vocabulary and system-based medical Spanish content covered during the course. Each question assessed vocabulary recognition, translation accuracy, and appropriate clinical phrasing across different organ-system domains (e.g., cardiopulmonary, gastrointestinal, neurologic). Each item was scored as correct/incorrect for a total possible score of 15 points.

After that, individuals were divided into groups based on proficiency (<30% on the quiz = beginner, 30-70% = intermediate, and >70% = advanced).

The course included a 10-week systems-based course where individuals learn associated terms for each system and complete two clinical scenarios, learning those terms in class. After completing the entire course, all students were given a post-course assessment and a post-course survey (Appendix F). Fourteen students participated in the study, allowing for a p < 0.05.

After the completion of this course, they repeated the same post-course assessment. As this was a locally developed pilot instrument, formal psychometric validation and reliability testing (e.g., Cronbach's alpha) were not performed. The exam was designed to reflect course learning objectives and parallel content covered in structured sessions. Students also filled out a five-question post-course survey.

Feasibility was determined by three domains: (1) recruitment (number of students enrolling relative to eligible class size), (2) attendance and completion rates across the 10-session curriculum, and (3) adequacy of sample size to permit exploratory statistical analyses of associations between attendance and comfort outcomes.

Given the pilot nature of the study, feasibility was defined as enrolling a sufficient number of participants to estimate effect sizes and detect statistically significant associations at α = 0.05 in exploratory regression models, recognizing that the study was not powered for definitive efficacy conclusions.

Data storage and statistical analysis

The only individuals with access to personal identifiers were study personnel in the WebIRB. Once the data were collected, it was stripped of personal identifiers, and each subject was given a particular code. These codified data were used to run simple linear regression models using RStudio (version 4.5.1; RStudio Team, Boston, MA).

Associations between session attendance and composite comfort scores were evaluated using simple linear regression models in R (RStudio, version 4.5.1). The primary model specified composite comfort score as the dependent variable and number of sessions attended as the independent variable. No additional covariates were included, given the small sample size and pilot nature of the study.

Regression coefficients (β), R² values, and two-sided p-values were reported for attendance analyses.

Pre- and post-course objective knowledge scores were compared using a paired t-test. Given the a priori directional hypothesis that scores would improve following course participation, a one-sided test was used for the paired analysis.

Missing data were handled using complete-case analysis. Only participants who completed both pre- and post-assessments were included in the paired objective knowledge analysis.

## Results

Group statistics

Fourteen students completed the surveys. Six participants were first-year students, seven were second-year students, and one was a third-year student.

Comfort measures

Self-reported comfort was assessed across five clinical Spanish domains using a 1-5 Likert scale (higher scores indicate greater comfort). Mean scores ranged from 2.54 to 3.54, indicating moderate overall comfort with substantial variability between individuals. These values can be seen in Table 1.

**Table 1:** Mean comfort in different interactions Scale: 1 = Very untrue; 2 = Somewhat true; 3 = Neither true nor untrue; 4 = Somewhat true; 5 = Very true

Item	Mean	SD
Introducing oneself	3.54	1.66
Performing interviews	3.00	1.47
Explaining procedures	2.85	1.68
Obtaining consent	2.54	1.61
Overall interaction with Spanish-speaking patients	3.38	1.39

A composite comfort score was created by averaging the five items for each respondent. The mean composite score was 3.06 (SD = 1.21), with values ranging from 1.8 to 5.0, suggesting moderate average comfort with variability across learners.

Associations among comfort domains and session attendance

Comfort measures were moderately to strongly intercorrelated. For example, comfort in introducing oneself correlated 0.78 with comfort performing interviews and 0.63 with overall comfort, while comfort explaining procedures correlated 0.80 with comfort obtaining consent. Correlations between individual comfort items and overall comfort ranged from approximately 0.27 to 0.63.

**Figure 1:**
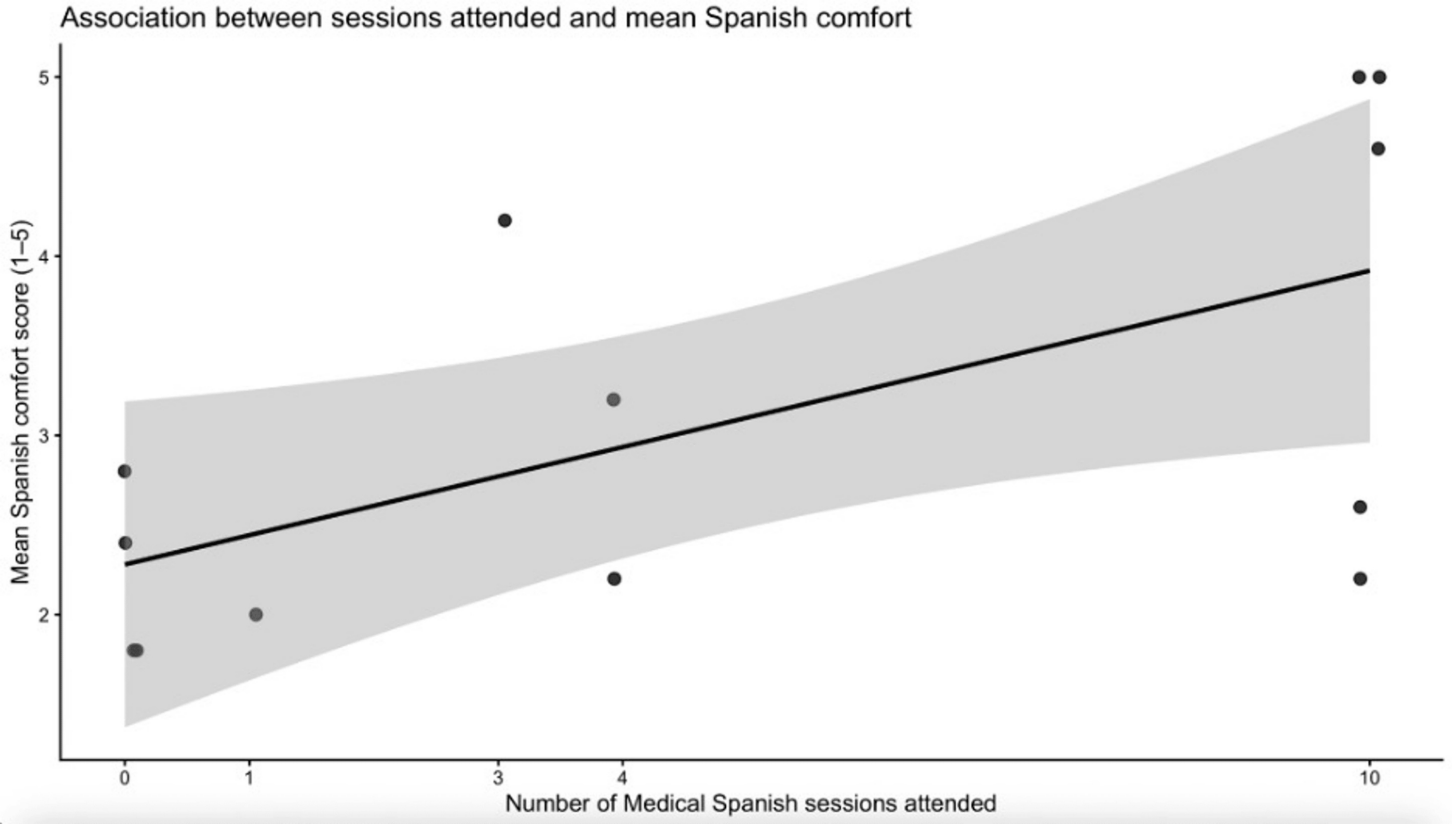
The association between the number of medical Spanish sessions attended and mean comfort speaking Spanish Intercept = 2.28: At 0 sessions, the mean overall comfort score is about 2.28 on the 1-5 scale. The slope for each session was 0.16 (p < 0.05). Each additional session attended is associated with about a 0.16-point increase in the mean comfort score, and this association is statistically significant at the 0.05 level in this small sample. Additionally, 37% of the variation in mean comfort is explained by the number of sessions. Scale: 1 = Very untrue; 2 = Somewhat true; 3 = Neither true nor untrue; 4 = Somewhat true; 5 = Very true The image was created by the authors using RStudio (version 4.5.1, GUI 1.82 High Sierra build (8536); Posit PBC, Boston, MA).

Composite comfort scores demonstrated a positive association with the number of sessions attended. Fourteen students completed the surveys, and increased session attendance was significantly associated with higher overall Spanish comfort (β = 0.16 points per session, p = 0.026; R² = 0.37), indicating a positive monotonic association in a small observational pilot. A positive trend was also observed for clinical-interaction comfort (β = 0.13), though this did not reach statistical significance (p = 0.16). Given the pilot nature and small sample size, these findings are interpreted cautiously (Figure 1).

Objective knowledge assessment

Objective assessment scores increased from a mean of 6.8 pre-course to 11.5 post-course (mean gain: 4.8 points). This improvement was statistically significant on paired analysis (t(3) = 3.31, one-sided p = 0.023) and demonstrated a large effect size (Cohen's d = 1.65).

Program evaluation framework

Using the Kirkpatrick model, this curriculum demonstrated evidence of learner satisfaction and knowledge acquisition (Levels 1-2). We classified the post-course Likert comfort/satisfaction survey as Kirkpatrick Level 1 (Reaction) and the objective pre-post-exam score change as Kirkpatrick Level 2 (Learning). Future studies with larger samples will be needed to evaluate behavioral change in clinical settings and patient-centered outcomes (Levels 3-4).

Objective assessment scores increased from a mean of 6.8 pre‑course to 11.5 post‑course (mean gain: 4.8 points), with a statistically significant improvement on a paired t‑test (one‑sided p = 0.023) and a large standardized effect size (Cohen's d = 1.65).

This study evaluated the curriculum using the Kirkpatrick model, demonstrating learner satisfaction and knowledge acquisition (Levels 1-2), with future work needed to assess behavioral transfer and patient-centered outcomes (Levels 3-4).

## Discussion

Background and implications

Despite the growing consensus that a medical Spanish curriculum should be included in medical schools, there is limited research as to what constitutes an effective program within a medical school. This study piloted a 10-session, system-based, and student-run course that incorporated translators and proficiency-based small groups, along with outside-of-class homework and learning resources, and evaluated efficacy with a pre- and post-course examination, along with a Likert scale to assess comfort speaking Spanish. Analysis of the data collected revealed that students' self-rated comfort speaking Spanish increased significantly with each additional lesson attended, in addition to an improvement in the pre- and post-course exams administered (d = 1.65). This pilot student-run medical Spanish program appears to be a feasible and promising model for future groups to build on, but results are highly preliminary, and further evaluation of this style of course is warranted.

The implementation of this peer-run medical Spanish program is one of the first of its kind to be evaluated in terms of not only the improvement in student comfort speaking Spanish but also the objective improvement in Spanish knowledge as measured by pre- and post-course examinations [10]. Both Martinez et al. and Oliver et al. are examples of peer-run medical Spanish programs at doctor of medicine (MD) schools; however, each of them evaluated the efficacy of the course with only student comfort speaking Spanish and post-course OSCE-style examinations [10,11]. While both do similarly show that student performance on post-course exams and OSCE met passing standards, there is no comparison that can be made to the student's baseline Spanish proficiency, and, thus, evaluation of the course's efficacy as a whole is limited [10,11].

Alternatively, studies like Highland et al. not only reported increased student comfort speaking Spanish, increased comfort in real clinical encounters with patients at a student-run free clinic in Colorado, but also revealed that Spanish-speaking patients' satisfaction scores equaled that of comparable patient satisfaction scores of English-speaking patients and providers [12]. Even though our course did not have the outcome of improved comfort in clinical settings, our study lacks the backing of a student-run free clinic in which students get to practice their Spanish speaking skills, which may limit our results. Future incorporation of OSCE-style practice or real practice at student-run free clinics may prove to be a very effective aspect of a medical school's Spanish curriculum.

One common factor between the course in this paper and the courses mentioned previously is that none used the same standardized exam to evaluate student efficacy in the pre- or post-course examination [13]. Moak et al. provided an example of a US MD school that implemented an Online Medical Spanish Course for its students during COVID, which was taught by professional Spanish teachers remotely [13]. This course was very effective at improving scores on the Cervantes Institute for the Diplomas of Spanish as a Foreign Language (DELE) exam based on pre- and post-course assessments [13]. While it is clear from all of the above studies that the courses improve both comfort speaking Spanish, as well as scores on post-course exams, future standardization of the metric used to do so is paramount in establishing the efficacy of these programs.

Curricular design

The curriculum was designed around in-person, student-run classes, which contributed to the strength of the course. Students were encouraged to practice while receiving real-time feedback from other students with prior Spanish training. Student leadership of the course supported learners through targeted instruction in language use, pronunciation, and cultural nuance. The systems-based structure allows student instructors to incorporate concepts learned in class into the medical Spanish curriculum, an approach that enhances integration of high-yield concepts with Spanish language and skills [11]. Another element that strengthened the curriculum was the proficiency-based grouping of students. Beginner students were instructed in separate groups with tailored content, allowing them to focus on foundational Spanish skills. Advanced students worked together to build their existing language proficiency. This approach reduced the workload for beginners while preventing boredom in advanced students, enabling both groups to achieve their learning objectives. These factors may increase student comfort in clinics, which has the potential to improve health outcomes for Latino patients [14].

Implications for training, equity, and trust

Studies have shown that miscommunication between patients and medical providers contributes to reduced satisfaction, quality of healthcare, and patient safety. Additionally, studies indicate that language barriers are the reason for partial understanding of patients' history and can lead to delayed treatment and mistreatment [15]. These factors can indirectly lead to increased healthcare costs. Other studies have also shown that most institutions have reduced access or no access at all to interpreter services [15]. Communication is the key component of the patient-provider relationship, and understanding and speaking a patient's language builds trust, improving long-term health outcomes. Students who introduce themselves and initiate a rapport in Spanish may help the patients feel more comfortable in the clinic [14]. Increased comfort can lead to improvements in history taking while reducing the reliance on interpreters.

Limitations

Despite the strengths, several limitations were considered. First, the sample size for the subjective assessment of competency included 14 students from California State University College of Medicine, while the objective assessment of competency included four students. These sizes limit the generalizability of the results and could indicate that the large effect size in our findings is unstable. Additionally, participation in the course was voluntary, which resulted in a self-selected group of students. These students may have had prior Spanish knowledge and intrinsic motivation to improve their proficiency. These factors contribute to the selection bias, limiting the generalizability of the findings to the broader population. Another limitation relates to the use of locally developed curriculum and assessment tools, which may limit the comparability of results across institutions. This introduces the possibility of limited external validity due to a single-institution design. Additionally, outcomes relied on self-assessment, raising the possibility that students evaluated their comfort or confidence rather than objective language proficiency. There was a potential Hawthorne effect where students may report increased comfort simply due to participation. Furthermore, students were grouped based on self-reported proficiency and comfort levels, rather than being randomized. This is a factor that could have influenced group learning outcomes. The absence of a control group limits causal inference. Finally, the one-sided p-value for the objective assessment may inflate statistical significance.

## Conclusions

In conclusion, our study provides preliminary evidence supporting the effectiveness of a student-run medical Spanish course in medical student education. We observed a clear dose-response relationship between session attendance and self-rated Spanish comfort, as well as significant improvements in objective pre- and post-examination scores. Although these findings are preliminary, this peer-led, in-person, proficiency-grouped curriculum represents a feasible and promising model for enhancing medical Spanish training in US medical schools. Broader implementation of similar curricula has the potential to strengthen patient-provider communication, reduce reliance on interpreters alone, and promote more equitable care for Spanish-speaking patients. Future research should include larger, multi-institutional studies using standardized assessment tools, as well as the incorporation of OSCEs or student-run free clinic encounters to better link learner gains with patient-centered outcomes.

## Figures and Tables

**Figure 2:**
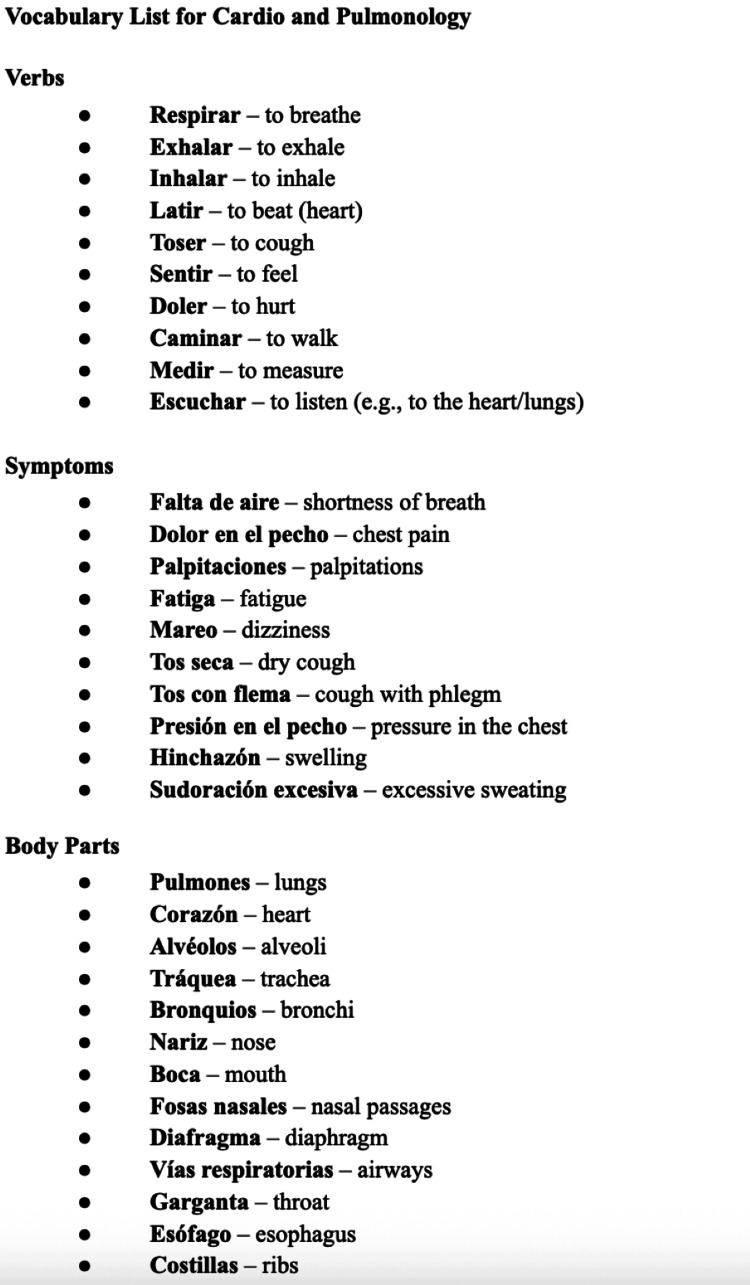
Example vocabulary list for cardio and pulm (Appendix B)

**Figure 3:**
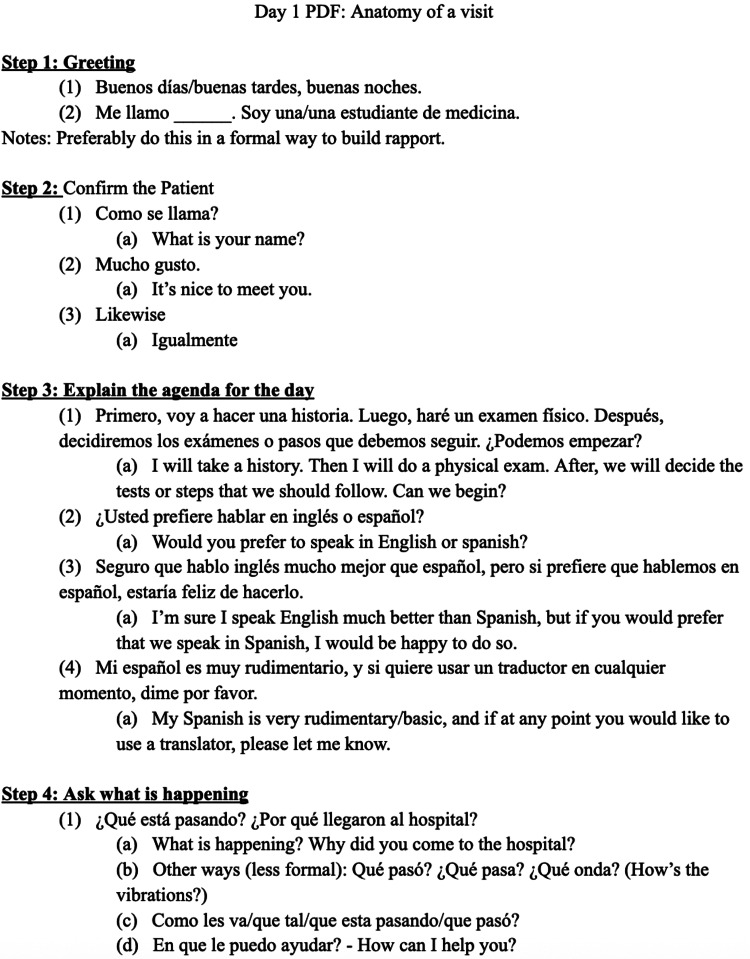
Day 1 worksheet (Appendix C)

**Figure 4:**
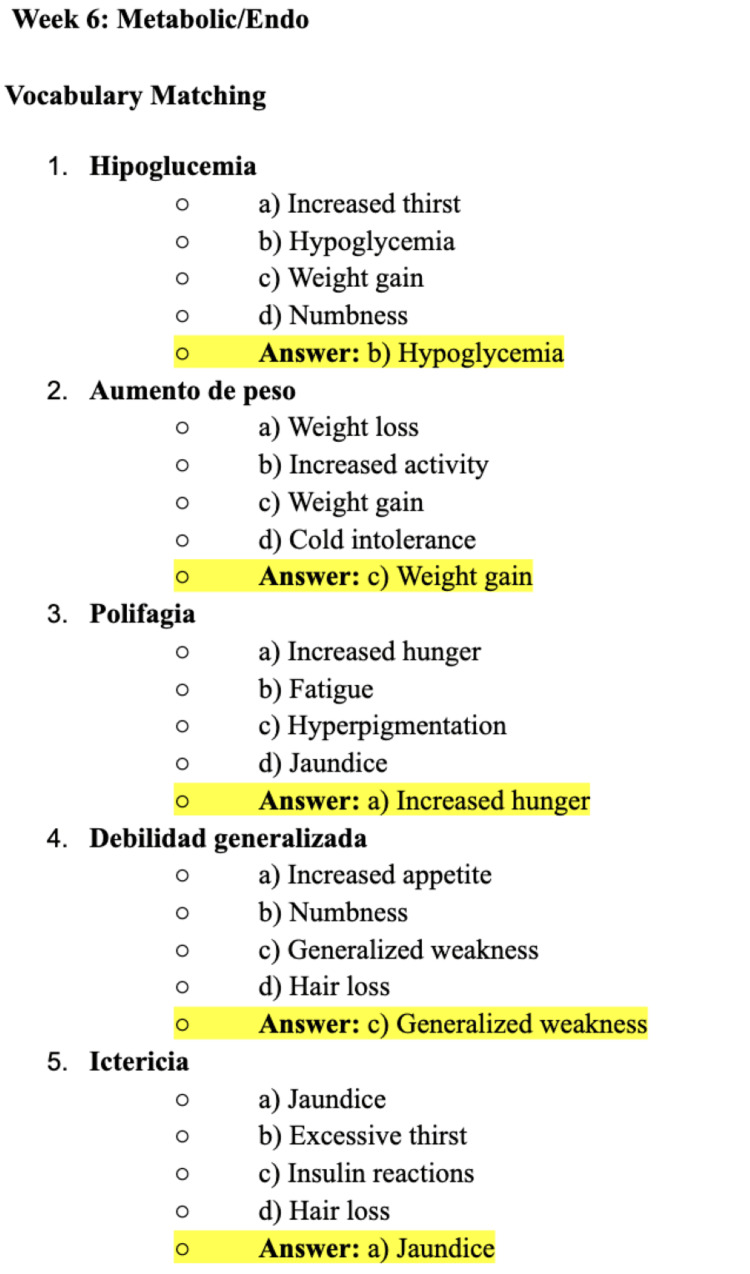
Example quiz (Appendix D)

**Figure 5:**
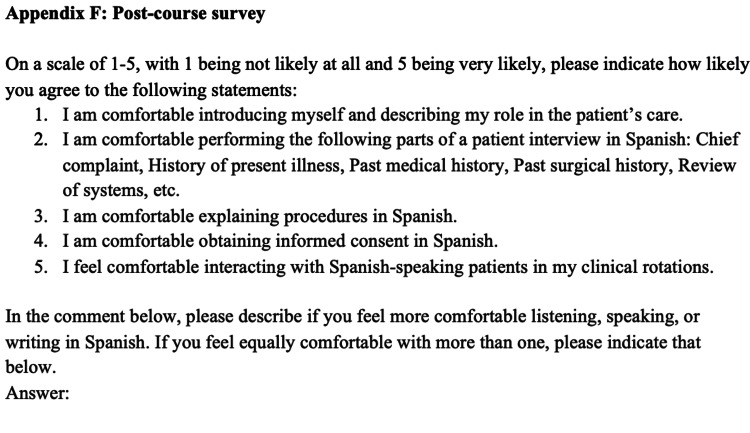
Post-course survey (Appendix F)

## Appendices

Appendices A-F present the medical Spanish course calendar (Table 2), example vocabulary list for cardio and pulm (Figure 2), day 1 worksheet (Figure 3), example quiz (Figure 4), pre-and post-course assessment (Table 3), and post-course survey (Figure 5).

**Table 2:** Medical Spanish course calendar (Appendix A)

Dates (subject to change)	Topic
Complete by Friday, 8/29/2025	Complete the pre-course assessment by 9/2/24 at midnight.
Thursday, 9/4/2025, 3:30-5 PM	Introduction to Medical Spanish: Pre-class Preparation | Presentation | Activity | Textbook PDF | Amazon Textbook Link | Session Summary | Vocabulary Textbooks: Spanish for Healthcare Workers.pdf, SPANISH Anatomy Medical Terminology.pdf
Thursday, 9/26/2024, 5:00-6:30 PM	Constitutional, External Body Parts, & Patient Intake Forms: Quiz #1 | Pre-Class Anki Deck | Presentation | Activity/Worksheet | Quiz for Beginners | Quiz for Intermediate and Advanced | Attendance Form
Thursday, 10/3/2024	Hematology: Pre-Class Vocab Sheet | Case (Student-Doctor Version) | Case 1 (Patient/ Preceptor Version) | Case 2 (Patient/ Preceptor Version)
Thursday, 10/24/2024	Musculoskeletal/Integumentary (Skin): Pre-Class Vocab Sheet | Quiz (beginners), Quiz Answers | Quiz (intermediate/Advanced) | Presentation
Wednesday, 10/30/2024	HEENT: Pre-Class Vocab Sheet | Presentation | Physical Examination Steps | Dia de los Muertos Cultural Competency Activity
Thursday, 11/14/2024	Metabolic/Endocrine: Quiz #3 | Pre-Class Vocab Sheet | Quiz (Beginners) | Quiz (Intermediate/Advance) | Attendance | Presentation | Clinical Case
Thursday, 12/5/2024	Gastrointestinal: Pre-Class Vocab Sheet | Attendance | Presentation | GI Lotería | GI Word Search | Clinical Case
Thursday, 1/9/2024	Neurological/Psychiatric: Pre-Class Vocab Sheet | Neurological Exam in Spanish | Attendance | Quiz #4: Quiz (Intermediate/Advanced), Quiz (Beginner) | Clinical Case | Presentation
Thursday 1/30/2024	Cardiovascular/Respiratory: Pre-Class Vocab Sheet | Attendance | Presentation | Cardiovascular and Respiratory System Vocabulary Practice Worksheet | Cardio/pulm Clinical Case
Thursday, 2/13/2024	Genitourinary/Reproductive: Pre-Class Vocab Sheet | Caso Clínico: Genitourinary | Genitourinary/Reproductive Presentation
Open Until 3/15/2024	Post-course assessment

**Table 3:** Pre-and post-course assessment (Appendix E)

Point	Question	Answer Choices	Answer Key
/0	Name		
/0	Email		
/0	Phone Number		
/0	Please indicate your prior Spanish-speaking knowledge.	1 year, 2 years, 3 years, 4 years, 4+ years. Native speaker, Second language, Other: ________	Exclusion criteria: native speaker
/0	A patient attends your primary care clinic with a headache. Please write below each step in their care.		
/1	1. Please choose the word that best fits the sentence: Hola, me llamo (your name) y soy _________. Patient: Hello, it is nice to meet you.	Manzana, Niño, Casa, Estudiante de medicina, No idea	Estudiante de medicina
/1	2. Please choose the word that best fits the sentence: You: ¿ ________ confirmar su nombre y día de nacimiento? Patient: My name is Jimenez Rodriguez and my date of birth is 5/1/1996.	Puede, Cómo, Cuantó, Qué, No idea	Puede
/1	3. Please choose the word that best fits the sentence: ¿ Qué ______ pasando? Jimenez: Tengo dolor de cabeza.	Es, Está, Comó, Tal, No idea	Está
/1	4. Please choose the word that best fits the sentence: ¿_______ ocurrió? Jimenez: Ocurrió hace cinco días.	Cómo, Cuando, Cuantó, Qué, No idea	Cuando
/1	5. Please choose the word that best fits the sentence: You: ¿ ____ tenido este problema anteriormente? Jimenez: Nunca. Es la primera vez.	He, Ha, Hemos, Hubo, No idea	Ha
/1	6. Please translate the following sentence: Can you describe where the pain is? Jimenez: The pain is localized to the left side of my head.	Free text	¿Puede describir dónde está el dolor?
/1	7. Please translate the following sentence: What happened when this pain began? Jimenez: I was walking, and the pain suddenly started and was preceded by flashing lights.	Free text	¿¿Qué pasó (o qué estaba pasando) cuando el dolor empezó?
/1	8. Please translate the following sentence: Does anything make it better or worse? Jimenez: Cuando estoy acostado en un cuarto oscuro, mejora el dolor, pero el estrés lo empeora.	Free text	¿Hay algo que lo mejore o lo empeore?
/1	9. Please translate the following sentence: You: Please describe the pain’s character and location. Jimenez: El dolor se irradia a través del lado izquierdo de mi cabeza y va y viene a lo largo del día.	Free text	Por favor, describa el carácter y la ubicación del dolor.
/1	10. Please translate the following sentence: You: Can you describe the pain on a scale of one to ten? (spell out the numbers in Spanish). Jimenez: Mi dolor es un ocho.	Free text	¿Puede describir el dolor en una escala del uno al diez?
/1	11. Please translate the following task (PMH). Ask the patient about their past medical history, including medications, hospitalizations, or surgeries. Response: The patient says that they were hospitalized for a tonsillectomy as a child, but otherwise have been healthy. They currently take Tylenol for headache pain.	Free text	Reader can understand the task.
/1	12. Please translate the following task (Social History): Ask the patient about their living situation, allergies, exercise, and diet. Response: The patient says that they live in a two-story apartment with their spouse, have no allergies, eat a “normal diet” and walk for 30 minutes per day.	Free text	Reader can understand the task.
/1	13. Please translate the following task (ROS): Please ask for at least 3 symptoms in the general/constitutional review of systems for the patient. Jimenez: No tengo ninguno de esos síntomas.	Free text	Reader can understand the task.
/1	14. Please translate the following task (Conclusion): Thank the patient, explain how you will go speak to the preceptor and return with a plan. Jimenez: Muchas gracias.	Free text	Reader can understand the task.
/1	15. Please listen to the following audio and choose the appropriate response. (Audio recording: Hola, gusto en conocerlo.)	No tengo nada, El gusto es mio, Está bien, Hasta mañana, De nada	El gusto es mio.
/0	Thank you for completing this assessment!		
